# Effects of perceived teacher support on online language learners’ engagement

**DOI:** 10.1016/j.heliyon.2024.e35679

**Published:** 2024-08-07

**Authors:** Lingqi Fan

**Affiliations:** a College of Foreign Languages, Nanjing University of Aeronautics and Astronautics, Nanjing, 210016, China; b Yandao Primary School, Chengdu, 610000, China

**Keywords:** Online language learner, Learner engagement, Perceived teacher Support

## Abstract

This study aimed to investigate the effects of perceived teacher support on online language learners’ engagement. Both quantitative and qualitative methods were adopted to explore the issue. A total of 1429 Chinese first-year non-English major students from different disciplines participated in the survey, and three of them were selected as semi-structured interviewees. The collected survey data were processed through multiple analyses with the help of SPSS 26.0 and AMOS 24.0, while qualitative data subsequently gathered from interviews served as supplementary evidence for examining the quantitative results. The findings indicated that online language learners were generally engaged at an intermediate level, and overall perceived teacher support was relatively close to the upper level. 10.13039/100014337Furthermore, online language learners’ engagement was positively correlated with perceived teacher support. Specifically, perceived intellectual support dominated in predicting overall engagement, cognitive engagement, emotional engagement, and social engagement, whereas perceived emotional support had the strongest influence on language learners’ behavioral engagement in the online context. The implications of these findings for future studies and educational practices were discussed.

## 1 Introduction

The early concept of learner engagement can be traced back to the 20th century, referring to *time on task*. Later, learner engagement is considered a meta-construct encompassing emotional, cognitive, and behavioral dimensions [1]. Motivated by this, researchers put much effort into investigating the multidimensional nature of learner engagement and gradually developed multiple conceptual frameworks of learner engagement [2,3]. As a key factor influencing academic achievement and assessing the learning process, learner engagement has continued to receive much attention from researchers both at home and abroad[[4], [5], [6]]. It plays an essential role in meaningful learning. It is argued that the efficacy of an educational programme or educational practice is directly linked to the result that whether it can enhance student engagement [7]. Only if a course generates adequate learner energy to further achieve certain learning outcomes can it exert the proposed effects. Therefore, it is of great necessity to understand how learners engage in their academic experiences.

Studies have shown that learner engagement is under the influence of various factors, including both internal and external factors, among which the teacher is considered to be a prominent one. Specifically, perceived teacher support is revealed to play a crucial role in positively affecting learner engagement in the previous studies [8,9]. In the digital age, online learning is an inevitable trend for future education, and COVID-19 did accelerate the development of online education. Nevertheless, the quick shift from the traditional learning context to an online learning context might potentially weaken the teacher-learner relationship due to less supervision and intervention caused by spatial distance. Whether and how perceived teacher support can exert impacts on learners’ engagement in the online context is rather vague. Only a small number of studies can be found in terms of examining the effects of perceived teacher support on online learners’ engagement [[10], [11], [12]].

In addition, studies on language learners’ engagement are still under-researched, as most of the researchers concentrate on learners from the general education field or other specific fields[[13], [14], [15]]. It is undeniable that language learners’ engagement is of equal necessity compared with learner engagement in other fields [16]. Learner engagement and perceived teacher support in language class are more subject-specific in light of the instrumental and linguistic duality of language teaching, and the association between the two variables cannot be directly deduced from research in the general educational field. Therefore, this research aimed at investigating the connections between perceived teacher support and language learners’ engagement in the online context.

## 2 Literature review

### 2.1 Learner engagement

The term e*ngagement* was initially germinated in the education field, as actively taking part in school activities could be defined as *engagement* [17]. Pioneers in exploring the connotations and measurements of learner engagement contended that student engagement contained both emotional engagement and behavioral engagement [18]. Afterwards student engagement has proven to be subjected to various interactive factors and can exert a certain influence on learning achievements[19,20]. The significance of learner engagement is self-evident, while researchers hold diverse views on its construct, hence developing a variety of constructs with different dimensions. A three-dimensional conceptual framework suggests that learner engagement should involve cognitive, behavioral and emotional dimensions [1], which is widely recognized and has prompted the creation of more comprehensive models of learner engagement in subsequent studies[2,3]. Learner engagement is a multidimensional meta-concept [1], which indicates that it is malleable and will be updated from time to time. Later in the language teaching context, engagement is considered as a state of high concentration and participation, and a four-dimensional engagement model (cognitive, behavioral, social, and emotional engagement) is constructed [21]. This four-dimensional model provides detailed definitions and explanations of each dimension, which brings valuable insights and references for promising empirical research. Based on the model, researchers have successively explored impact studies on language learners’ engagement[22,23].

Online learning is an unavoidable trajectory for future education in the digital era, and the pandemic has expedited its progress. The epidemic prevented 98 % of the global student population, which amounts to over one billion students, from studying offline [24]. Schools and institutions were compelled to rapidly shift from face-to-face classes to remote education, which brought challenges for both educators and students. One notable consequence is that the teacher-student relationship has weakened as the spatial distance has lengthened. Traditional teaching context enables the teacher to precisely sense and supervise whether learners are engaged in class through their facial expressions and behaviors, while it is inapplicable for online teaching context [25]. Additionally, distant education may impede learners from having timely interaction with their peers or instructors [10], particularly in the context of language learning, which is highly interactive. Therefore, how to accurately assess the quality of online education has become a pivotal concern. Learner engagement is a significant indicator for assessing learning efficiency, and researchers have explored the nature of learner engagement in the online context. Many researchers agree that online learner engagement measures the effort learners put into classroom activities to achieve certain learning outcomes[[26], [27], [28]]. The dominant view suggests that online learner engagement is similar to offline learner engagement and is not confined to an uni-dimensional concept but involves multiple dimensions[29,30]. Noticeably, even though learner engagement is conceptualized as varied constructs, most researchers found that learners engaged moderately in both traditional and online context[[31], [32], [33], [34]], only limited research concluded that learners engaged at a high level [35].

On the basis of the aforementioned views, this study defines online language learners’ engagement as consisting of cognitive, behavioral, emotional, and social engagement. Cognitive engagement requires sustained attention, energy, and mental effort from online language learners, and also involves focusing on language forms [36]. Specific learning behaviors that students actively display both inside and outside of the classroom are known as behavioral engagement. Emotional engagement, learners’ reaction that promotes their language learning engagement, is often expressed in emotions such as pleasure, anticipation, enthusiasm, enjoyment, and so forth[[37], [38], [39]]. Social engagement focuses on learners’ social participation during the learning process, which reflects the interactive nature that is unique to language learning and emphasizes the connections between learners and peers, the learning materials as well as the instructor.

### 2.2 Perceived teacher support

Teachers are the people with whom students form social bonds in school and serve as a crucial source of support for learners. Currently, one dominant view of what constitutes teacher support is founded on social support theory. Social support refers to the care, affection, and respect that an individual receives from peers, family, community, colleagues, teachers and so on [40]. In simple terms, social support includes several forms such as peer support, parental support, teacher support and so on. There is a widely held belief that learners are unable to fully perceive all of the support that teachers offer, which results in an inherent discrepancy between provided teacher support and perceived teacher support. Most researchers at home and abroad stick to the view that only when teacher support is perceived by learners can it exert an impact on them, and research on perceived teacher support is of higher value than provided teacher support[41,42]. Therefore, this study also concentrates on teacher support from learners’ perspectives.

Perceived teacher support has been defined by many scholars as emotional, instrumental, informational, and appraisal support [41,43,44] based on the social support views of Tardy and House[45,46], while some researchers have drawn on part of these ideas to conceptualize perceived teacher support into emotional and instrumental dimensions[47,48]. Jiang et al. [42] proposed a four-dimensional model of perceived teacher support in online educational settings on the basis of previous studies, which consists of emotional support, instrumental support, intellectual support, and social support. Emotional support pertains to the favourable sentiments that learners acquire during their learning experiences. Instrumental support refers to the guidance and assistance that teachers provide to learners. Intellectual support focuses on teachers’ knowledge, skills, experience, and strategies. Social support is the support that teachers provide to learners in the classroom through teacher-student interaction or peer interaction. Generally, there are several overlaps between this conceptual model and earlier models, whereas social support is a newly-added dimension.

The definition of perceived teacher support varies according to specific research contexts and objectives. Additionally, different teaching modes may lead to adaptive modifications in the interpretation of concepts, and there are also subject-specific differences among academic disciplines, which indicates that learners may require varying forms of teacher support in diverse learning contexts. What can be guaranteed, though, is that teacher support is a multidimensional meta-concept and is malleable across various teaching scenarios[42,47]. While yet to be established as a unified concept in the pertinent literature, perceived teacher support undeniably holds considerable importance and it has been measured accordingly, be it from overall or single-dimension level[35,49]. Studies have reported overall perceived teacher support at both high level[50,51]and intermediate level[34,44,52,53] due to varied measurements, sample size, demographic characteristics etc. Furthermore, the burgeoning body of related research has found that perceived teacher support has multifaceted effects on learners’ learning processes, including academic enjoyment, academic achievement, motivation, self-efficacy, and so on[12,35,43,54,55].

This study adopts the framework (emotional, instrumental, intellectual, and social support) [42] to define perceived teacher support in the online language teaching context. For one thing, this definition, which has been acknowledged and embraced by prior research[56,57], specifically targets at online educational settings. For another, it incorporates the social aspect of interaction between students and instructors, which aligns with the interactive nature of language learning and online teaching.

### 2.3 Relationships between perceived teacher support and learner engagement

In the ecosystem model of factors influencing learner engagement constructed by Bond & Bedenlier [58], teachers are identified as the factor in the first circle (micro system). Undeniably, teachers play a crucial role in supporting learners in school and are able to exert profound influence on all aspects of learner development. A number of empirical studies have verified that perceived teacher support has a positive predictive effect on learner engagement in traditional teaching contexts, regardless of whether it is assessed at an overall level, a single dimension, or multiple dimensions[28,54,59,60]. Online educational settings separate learners and educators geographically, which possibly further resulting in a hindrance to the exchange of knowledge, a decrease in emotional satisfaction, and a rise in loneliness and anxiety[61,62]. In this case, the demand for teacher support is greater and necessitates a wider range of aspects compared to the traditional classroom setting. Only several studies have proven that perceived teacher support in the online context could significantly predict learner engagement[10,55,63], which explored a single dimension of the two factors rather than taking full perspectives.

In addition, while impact studies have validated the connection in the general education field[8,15,64], whether perceived teacher support can exert influence on language learner engagement still needs to be further explored. Mercer & Dornyei [16] claim that learner engagement in language teaching is just as vital as in other subjects. Language is fundamentally distinct from other disciplines, such as mathematics and physics. For one thing, language teaching prioritizes the instrumental function, which requires learners to use a foreign language to solve problems in class, whereas learners in other disciplines are able to solve problems in their familiar mother tongue. For another, the foreign language serves as a medium for teachers to support and interact with students, and also as teaching content, which urges teachers to pay close attention to both students’ linguistic output and theirs. Therefore, learner engagement and perceived teacher support are more discipline-specific when it comes to the language teaching field, so the relationship between the two variables cannot be directly deduced from research in general education. The previous empirical evidence showed that social, instrumental, and autonomy support from teachers in foreign language teaching classrooms had a positive impact on learner engagement [65]. In the study conducted by Sadoughi & Hejazi [43], the findings indicated a positive and direct correlation between teacher support and academic engagement, but specific predicting roles were not identified.

However, there are still a few conflicting views related to impact studies. Some researchers revealed that perceived teacher support did not correlate with learner engagement[66,67]. Han, Geng and Wang [65] conducted an empirical study among 428 university English learners in an online context and found that perceived teacher support was negatively associated with learners’ affective engagement and behavioral engagement and that students who perceived more emotional and instrumental teacher support had lower emotional engagement, which was presumably due to the fact that the teacher failed to give enough autonomy support to the students and that too much instrumental support could reversely be an interference that hindered the satisfaction of the autonomy needs of the students. Thus, the correlation of the two variables needs to be further confirmed. To the best of our knowledge, no research can be found in terms of investigating the relationship between online language learners’ engagement (cognitive, emotional, behavioral, and social engagement) and perceived teacher support from emotional, instrumental, intellectual, and social aspects.

### 2.4 Research questions

Derived from the literature review, this study employed both quantitative and qualitative approaches and aimed to explore three research questions: (1) To what extent do language learners cognitively, behaviorally, emotionally, and socially engage in online College English course? (2) To what extent do language learners perceive instrumental, emotional, intellectual, and social support from the teacher in online College English course? (3) What are the effects of perceived teacher support on language learner engagement in online College English course? Based on previous research, this study hypothesized that perceived teacher support would positively predict online language learners’ engagement.

## 3 Methods

### 3.1 Research design

This study adopted explanatory sequential methods. When methods are combined, quantitative and qualitative approaches complement each other and may offer new perspectives and more evidence for research results when integrating two forms of data [68]. Firstly, the research collected and analyzed quantitative data. To elucidate students’ responses to engagement and perceived teacher support, an online questionnaire was utilized to test students’ reactions with two 5-point Likert scales. A typical university was selected as the sample. The questionnaires were designed and handed out via an online questionnaire application. A pilot study was conducted after participants had completed weeks of online courses. Then, formal questionnaires were further distributed at the end of the semester. Secondly, the subsequent qualitative phase followed in order to validate and expand the previous quantitative results. Specifically, semi-structured interviews were conducted after the survey. The interview questions were designed as open-ended questions to find out whether online language learners perceived teacher support and how their perceived teacher support could exert an impact on learning engagement. Data collected from the interviews served as supplementary evidence to complement the reliability of the survey results, thus maximizing the theoretical and practical value of the research.

### 3.2 Sample

The overall survey participants engaged in this research were 1849 first-year non-English major students from different disciplines recruited from University X in China, and 1429 participants (Science 23.65 %, Engineering 20.85 %, Medicine 18.05 %, Law 8.12 %, Education 7.70 %, Management, 7.07 %, Literature 5.11 %, Economics 4.76 %, Agricultural, 3.08 %, Art, 1.33 %), with males (39.68 %) and females (60.32 %), were identified as valid. Therefore, the valid response rate reached 77.29 %.

### 3.3 Research procedures

To answer research questions, addressing the status quo of online language learners’ engagement, perceived teacher support and their connections, this research first adopted survey method which dominates in measuring learner engagement and perceived teacher support. Its high adaptability renders it suited for various educational settings and multiple types of learners. Due to the ongoing COVID-19 pandemic, students at the sample university were required to take online courses for nearly the whole semester. Therefore, University X was the tailored choice for this research to recruit online language learners. By and large, participants can be classified into questionnaire participants and interview participants.

Initially, a pilot study (N = 269) was conducted to ensure the reliability and validity of the adapted scales. After the revision of scales, formal study ensued. In order to efficiently cleanse the data, it was necessary to exclude some questionnaires with the same response for every item and short filling time. At the beginning of the semester, the first-year students were divided into classes of varying levels, namely Level A (high proficiency), Level B (intermediate proficiency), and Level C (low proficiency), based on their English Test scores from College Entrance Examination. The participants in this study were from both Level A and Level B, indicating that they had a relatively good command of English language. Valid participants engaged in this survey were 1429 first-year non-English major students from different disciplines.

In the next stage, to further understand the effects of perceived teacher support on language learners’ engagement in the online teaching context, semi-structured interviews were conducted with three participants chosen from the formal survey pool. It is of great significance to investigate comprehensive perspectives from learners of different engagement levels. Participants were divided into three levels, namely high (top 27 %), intermediate and low (last 27 %), based on the preliminary results of the survey. Then, one participant from each level were selected at random to act as interview subjects(N = 3). Instructions and questions were stated in Mandarin to make sure that participants could fully understand and then freely articulate their feelings. Each interview lasted about 25 min and was audio-recorded with participants’ consent.

### 3.4 Instrument

This study employed two scales: O*nline Language Learners’Engagement Level Scale* and *Online Language Learners’Perceived Teacher* Support *Level Scale*. The first scale was designed based on *the Online Engagement in Higher Education Scale* [69], *English Learner Engagement Scale* [70], *Online Language Learning Scale* [51] and *MOOC Learners’Engagement Scale* [71]. The final *Online Language learners’Engagement Scale* was a 5-point Likert scale that consisted of four dimensions, including cognitive engagement, behavioral engagement, emotional engagement and social engagement, and there were 17 items in total. Participants could simply respond to each item in line with their own opinions on a 5-point Likert scale ranging from 1 = completely disagree, 2 = disagree, 3 = unsure, 4 = agree to 5 = completely agree. Cronbach's alpha values in each dimension and in the overall scale were 0.867, 0.746, 0.867, 0.785 and 0.908 respectively, which indicated a high level of reliability, and the data from confirmatory factor analysis showed the high validity of the scale (CMIN/DF = 4.034, RMSEA = 0.046, RMR = 0.018, NFI = 0.961, CFI = 0.970). According to Wu (2013), the CMIN/DF value is highly responsive to changes in sample size. In the context of large-sample research, the probability for the figure to fall inside the interval of 0–3 is quite slim. In this case, the CMIN/DF value no longer serves as the relevant indicator for model fit, while other indicators such as RMSEA, RMR, NFI, CFI and so on together offer comprehensive assessment.

Measurements of perceived teacher support in language classes are relatively limited, let alone in the online educational setting. *Online Language Learners’Perceived Teacher Support Scale* for this research was designed on the basis of the *Online learners’Perceived Teacher Support Scale* [42], *Perceived Social Support-English Learning survey* [51], *Class Life Measure*[72] and *Instrumental Support Scale* [73]. The designed scale was composed of four dimensions, including emotional support, instrumental support, intellectual support and social support, and there were 19 items in total. This scale was also in the form of a 5-point Likert scale ranging from 1 = completely disagree, 2 = disagree, 3 = unsure, 4 = agree to 5 = completely agree. Cronbach's alpha values for each dimension and the whole scale were 0.907, 0.794, 0.904, 0.876 and 0.941 respectively, which demonstrated high reliability, and the data from confirmatory factor analysis ensured the high validity (CMIN/DF = 3.761, RMSEA = 0.044, RMR = 0.011, NFI = 0.968, CFI = 0.977).

### 3.5 Data analysis

Quantitative analysis started with descriptive analysis to answer Research Question 1 and 2. In this way, indicators such as means, percentage, standard deviation and so forth, would provide general information regarding online learners’ engagement and perceived teacher support. Next, independent samples T-test and one-way ANOVA were performed to delve deeper into Research Question 1 and 2 with additional information on whether differences, particularly demographic characteristics, exist in individual learner’s engagement and perceived teacher support. Finally, Research Question 3 were addressed by employing correlation analysis and multiple linear regression analysis respectively.

Qualitative analysis concentrated on the data collected from the semi-structured interviews. Data processing steps closely followed the suggestions from John Creswell [74]. First of all, audio recordings were transcribed into text. Secondly, after reading the texts, the author recognized, categorized, and coded the meaning units that were pertinent to the research questions. Then, conclusions were developed based on the codes and later stated in the current work as extra supporting evidence responding to Research Question 3.

## 4 Results

### 4.1 Online language learners’ engagement

The results of the descriptive analysis can be found in Table 1, which indicated a medium level of overall online language learners’ engagement (M = 3.660, SD = 0.489). In terms of levels of different dimensions, social engagement (M = 3.765, SD = 0.541) took the lead, closely followed by behavioral engagement (M = 3.716, SD = 0.601), cognitive engagement (M = 3.637, SD = 0.616) and emotional engagement (M = 3.565, SD = 0.686). With regard to specific items in each dimension, SE3 (I try to do the tasks with classmates who can help me) was the top one item (M = 3.840, SD = 0.648), closely followed by SE1 (I try to help the students who meet with difficulties when learning online) (M = 3.800, SD = 0.666), while SE2 (*I like working with others to finish tasks in online English class*) got the lowest mean (M = 3.670, SD = 0.755). As for behavioral engagement dimension, BE3 (*I complete the class-related practice/homework*) ranked the highest in the item list (M = 3.950, SD = 0.648), whereas BE1 (*I learn English outside of the online English class*) got the lowest mean (M = 3.490, SD = 0.844). CE3 (*I try to figure out how the knowledge learned in online English class might be used in the real world*) (SD = 0.746) and CE5 (*I reflect on my understanding of the feedback that I get in the online English class*) (SD = 0.721) were scored the highest on average (M = 3.700). It can be deduced that most of the online language learners engaged themselves on a deeper level cognitively. It is worth mentioning that CE2 (*I try to set examples to better understand the important concepts in online English class*) had the lowest mean (M = 3.520, SD = 0.805), which could be inferred that online language learners held different views on their knowledge comprehension approach. For emotional engagement, EE2 (*I enjoy learning new things in online English class*) achieved the highest score (M = 3.720, SD = 0.787), whereas EE4 (*I enjoy sharing my opinions/experiences with the teacher and classmates in online English class*) obtained the fewest votes (M = 3.430, SD = 0.889).

**Table 1:** Descriptive analysis of online language learners’ engagement.

Dimension	M	SD
Cognitive Engagement	3.637	0.616
Behavioral Engagement	3.716	0.601
Emotional Engagement	3.565	0.686
Social Engagement	3.765	0.541
Overall Engagement	3.660	0.489

Emotional Engagement	3.565	0.686
Social Engagement	3.765	0.541
Overall Engagement	3.660	0.489

This study utilized independent sample T-test and one-way ANOVA to explore the individual differences in online language learners’ engagement with regard to demographic variables. Independent sample T-test results suggested that there was no significant difference between male and female language learners in cognitive engagement, behavioral engagement, emotional engagement and social engagement respectively (p = 0.930 > 0.05, p = 0.502 > 0.05, p = 0.938 > 0.05, p = 0.604 > 0.05). The results of one-way ANOVA indicated that no significance could be found among different disciplines in terms of four dimensions of online language learners’ engagement (p = 0.158 > 0.05, p = 0.606 > 0.05, p = 0.116 > 0.05, p = 0.090 > 0.05).

### 4.2 Perceived teacher support

A descriptive analysis was conducted to unveil the current situation of perceived teacher support. The data presented in Table 2 indicated that online language learners generally perceived an upper-middle level of teacher support during their online study (M = 3.978, SD = 0.440). In terms of four dimensions, intellectual support ranked the highest (M = 4.033, SD = 0.506), followed by emotional support (M = 4.017, SD = 0.520), instrumental support (M = 4.017, SD = 0.510), and social support (M = 3.849, SD = 0.563). Specifically in intellectual support dimension, InteS2 (*Teacher gives lesson with clear pronunciation, fluent expression in online English class*) dominated (M = 4.080, SD = 0.590). As for emotional support, ES4 (*Teacher respects my response and feedback in online English class*) had the highest average score (M = 4.070, SD = 0.567). In terms of instrumental support, InstS2 (*Teacher provides me with useful guidance and help in online English class*) ranked first (M = 4.070, SD = 0.587). With regard to social support dimension, SS3 (*Teacher notices my statements in the chat box or on other platforms, and gives response*) took the lead (M = 3.950, SD = 0.628).

**Table 2:** Descriptive analysis of online language learners’ perceived support.

Dimension	M	SD
Emotional Support	4.017	0.520
Instrumental Support	4.017	0.510
Intellectual Support	4.033	0.506
Social Support	3.849	0.563
Overall Support	3.978	0.440

Independent sample T-test and one-way ANOVA were used in this study to examine how demographic characteristics affected online language learners’ perceived teacher support. The results revealed that there was no significant difference between male and female participants in perceived emotional support, instrumental support, intellectual support and social support respectively (p = 0.721 > 0.05, p = 0.100 > 0.05, p = 0.689 > 0.05, p = 0.165 > 0.05). In addition, results of one-way ANOVA showed that p values for all dimensions were larger than 0.05, demonstrating that there was no significant difference among different disciplines regarding perceived teacher support in online English classes.

### 4.3 Effects of perceived teacher support on online language learners’ engagement

In this section, both quantitative and qualitative results of associations between online language learners’ engagement and perceived teacher support would be presented respectively through the results of correlation analysis, multiple linear regression analysis and semi-structured interviews.

#### 4.3.1 Correlation analysis

As is shown in Table 3, the correlation between learners’ overall engagement and overall perceived teacher support was statistically significant (p < 0.01). The Pearson correlation coefficient was 0.616, which revealed a positive relationship between the two variables. In addition, the Pearson correlation coefficient within the range of 0.4–0.7 indicates moderate correlation, while the range of 0–0.4 shows low correlation [75]. Thus, it can be concluded that learners’ engagement was positively and moderately correlated with perceived teacher support. With regard to specific dimensions, a positive correlation also existed to a different extent. Therefore, further multiple linear regression analysis could be conducted to thoroughly examine the specific relationship between the two variables in detail.

**Table 3:** Pearson correlation analysis result.

Dimension	Cognitive Engagement	Behavioral Engagement	Emotional Engagement	Social Engagement	Overall Engagement
Intellectual Support	0.382**	0.408**	0.476**	0.611**	0.585**
Social Support	0.356**	0.351**	0.409**	0.445**	0.493**
Instrumental Support	0.319**	0.281**	0.336**	0.353**	0.409**
Emotional Support	0.346**	0.426**	0.432**	0.513**	0.533**
Overall Perceived Support	0.424**	0.449**	0.505**	0.591**	0.616**

Note. **p<0.01

#### 4.3.2 Multiple linear regression analysis and semi-structured interviews

In this part, multiple linear regression analysis was performed through a stepwise approach to explore the effects of perceived teacher support on online language learners' engagement and statistical models were established respectively to present the specific association between variables, hence addressing the Research Question 3. Additionally, the results of semi-structured interviews would be reported as supplementary evidence for the effects of perceived teacher support on online language learners’ engagement.

(1) Overall effect of perceived teacher support upon online language learners’ engagement

First of all, the overall effect of perceived teacher support on online language learners’ engagement were explored as in Table 4. The predictors included perceived social support, emotional support, intellectual support, instrumental support, and the dependent variable was overall online language learners’ engagement. In total, four models (p = 0.000) were tested as significant through a stepwise process and among which the most predictive one Model 4 (R^2^ = 0.404) contained four predictors.

**Table 4:** Summary of model^e^ parametric test.

Model	R	R Square	Adjusted R Square	Std. Error of the Estimate	F	Sig.
1	0.585^a^	0.343	0.342	0.397	744.241	0.000
2	0.630^b^	0.397	0.396	0.380	468.577	0.000
3	0.634^c^	0.402	0.401	0.379	319.384	0.000
4	0.635^d^	0.404	0.402	0.378	241.117	0.000

a . Predictors: (constant), Intellectual Support.

b . Predictors: (constant), Intellectual Support, Emotional Support.

c . Predictors: (constant), Intellectual Support, Emotional Support, Social Support.

d . Predictors: (constant), Intellectual Support, Emotional Support, Social Support, Instrumental Support.

e . Dependent Variable：Overall Online Language Learners' Engagement.

As stated in Table 5, the p-values for each predictor in Model 4 were below, which denoted that every independent variable could significantly predict the dependable variable. VIF examines the multi-collinearity among different variables. According to Wu [76], VIF value within the range of 0–10 can be considered a sign of no significant multi-collinearity existing in independent variables. The statistics indicated that the VIF values for intellectual support, emotional support, social support, and instrumental support all fall within the acceptable range (VIF = 1.748, VIF = 2.225, VIF = 2.259, VIF = 1.807). Therefore, there was no severe multi-collinearity found in Model 4.

**Table 5:** Detailed information of regression model of overall online language learners’ engagement.

Model		Unstandardized Coefficients		Standardized Coefficients	t	Sig.	Collinearity Statistics	
		B	Std. Error	Beta			Tolerance	VIF
4	(constant)	1.018	0.093		10.987	0.000		
	Intellectual Support	0.331	0.023	0.381	14.095	0.000	0.572	1.748
	Emotional Support	0.204	0.029	0.217	7.108	0.000	0.449	2.225
	Social Support	0.082	0.030	0.085	2.774	0.006	0.443	2.259
	Instrumental Support	0.054	0.026	0.056	2.044	0.041	0.553	1.807

Dependent Variable：Overall Online Language Learners’ Engagement.

Based on the detailed statistics, the standard regression equation of overall online language learners’ engagement can be established as Y = 0.381X_1_ (intellectual support) + 0.217X_2_ (emotional support) + 0.085X_3_ (social support) + 0.056X_4_ (instrumental support), which illustrated the existence of a positive linear relationship between the dependent variable and the independent variables. The equation implied that perceived intellectual support and emotional support mainly affected the overall online language learners’ engagement, while perceived social support and instrumental support had a marginal impact on the dependent variable.

Responses collected from the semi-structured interviews showed similar results, which validated the quantitative analysis results. Interview questions 1 and 2 were related to the effects of perceived teacher support on overall learners’ engagement. From the response of interviewee A, it can be found that perceived emotional support, social support and intellectual support from the teacher had a significant impact on her overall engagement, as she specifically mentioned that the teacher’s knowledge reservoir, encouragement and available resources in online English class could encourage and motivate her to learn. In terms of interviewee B, she notably highlighted that the teacher’s tactics and timely assistance boosted her English learning. This underscored the influence of instrumental and intellectual support on the learner’s overall engagement. Interviewee C pointed out his perceptions of emotional support, social support and intellectual support from the teacher through encouragement, communication, provided guidance and resources. Nevertheless, he mentioned that perceived teacher support failed to have prolonged impacts on his engagement (*I think the support from the teacher may help us engage in class to some degree. But it is temporary, and I would sometimes be absent-minded still*).

Overall, both quantitative and qualitative analysis results witnessed a significant influence of perceived teacher support, especially intellectual support and emotional support, on overall online language learners’ engagement. Therefore, the hypothesis was accepted.

(2) Effect of perceived teacher support upon online language learners’ cognitive engagement

Specific effect of perceived teacher support on online language learners’ cognitive engagement was investigated through a stepwise approach. As shown in Table 6, four significant models (p < 0.05) were achieved and Model 4 was the most predictive model (R^2^ = 0.182) in comparison to its counterparts, including all four dependent variables.

**Table 6:** Summary of model^e^ parametric test.

Model	R	R Square	Adjusted R Square	Std. Error of the Estimate	F	Sig.
1	0.382^a^	0.146	0.145	0.570	243.991	0.000
2	0.417^b^	0.174	0.173	0.561	149.933	0.000
3	0.426^c^	0.182	0.180	0.558	105.436	0.000
4	0.430^d^	0.185	0.182	0.557	80.552	0.000

a . Predictors: (constant), Intellectual Support.

b . Predictors: (constant), Intellectual Support, Instrumental Support.

c . Predictors: (constant), Intellectual Support, Instrumental Support, Social Support.

d . Predictors: (constant), Intellectual Support, Instrumental Support, Social Support, Emotional Support.

e . Dependent Variable：Cognitive Engagement.

With regard to the detailed model information shown in Table 7, the p values for each independent variable in Model 4 were lower than 0.05, which suggested that these variables had a significant impact on predicting learners’ cognitive engagement. Additionally, VIF values were exactly within the reasonable range, providing further proof for the model’s efficiency.

**Table 7:** Detailed information of regression model of online language learners’ cognitive engagement.

Model		Unstandardized Coefficients		Standardized Coefficients	t	Sig.	Collinearity Statistics	
		B	Std. Error	Beta			Tolerance	VIF
4	(constant)	1.283	0.137		9.393	0.000		
	Intellectual Support	0.248	0.035	0.226	7.153	0.000	0.572	1.748
	Instrumental Support	0.131	0.039	0.108	3.364	0.001	0.553	1.807
	Social Support	0.123	0.044	0.101	2.806	0.005	0.443	2.259
	Emotional Support	0.095	0.042	0.080	2.239	0.025	0.449	2.225

Dependent Variable：Cognitive Engagement.

With Beta coefficients in the detailed regression model, a standard regression equation of online language learners’ cognitive engagement can be concluded as Y = 0.226X_1_ (intellectual support) + 0.108X_2_ (instrumental support) +0.101X_3_ (social support) + 0.08X_4_ (emotional support). According to the equation, perceived intellectual support dominates in predicting online language learners’ cognitive engagement, while emotional support has a relatively minimal effect on it.

Data collected from semi-structured interviews also indicated that online learners’ engagement was under the influence of four dimensions of perceived teacher support. Interviewee A mentioned that perceived intellectual support and social support facilitated her cognitive engagement as she would like to pay more attention to the class in light of the professionalism of the teacher and well-organized interactive activities. In addition, she said that provided scaffolding in speaking activities was conducive to knowledge application, which highlighted the function of perceived instrumental support. At last, she also expressed that the positive appraisal from the teacher promoted the feedback reflection. In other words, interviewee A believed that her cognitive engagement was positively influenced by perceived intellectual support, social support, instrumental support and emotional support. For interviewee B, she thought that perceived intellectual support, instrumental support and social support had positive effects on cognitive engagement. To be specific, she mentioned that the teacher’s professional competence, personal charm and provided interactions could attract her attention to help her focus on the class. Interviewee C contended that perceived instrumental support was likely to improve online language learners’ engagement. Given that interviewee C provided ambiguous answers to the questions, there was a dearth of evidence for cognitive engagement promotion, which was presumably caused by the low engagement of the interviewee.

In conclusion, evidence from quantitative and qualitative analysis showed that online language learners’ engagement was determined by perceived social support, instrumental support, intellectual support and emotional support.

(3) Effect of perceived teacher support upon online language learners’ behavioral engagement

Through a stepwise approach, the effect of perceived teacher support on online language learners’ behavioral engagement were examined and findings are shown in Table 8. Two significant models (p < 0.05) were derived from the regression analysis. Model 2, the most predictive one (R^2^ = 0.218), included both emotional and intellectual support, explaining 21.8 % of the variance in the behavioral engagement of distant language learners. Evidence clearly demonstrated that perceived instrumental support and social support did not significantly influence learners’ behavioral engagement in remote language education environments.

**Table 8:** Summary of model^c^ parametric test.

Model	R	R Square	Adjusted R Square	Std. Error of the Estimate	F	Sig.
1	0.426^a^	0.181	0.181	0.544	316.002	0.000
2	0.468^b^	0.219	0.218	0.531	200.099	0.000

a . Predictors: (constant), Emotional Support.

b . Predictors: (constant), Emotional Support, Intellectual Support.

c . Dependent Variable：Behavioral Engagement.

According to the data from the regression model in Table 9, each predictor in Model 2 had a p value that was lower than 0.05, which implied that each independent variable had a considerable chance of predicting the dependent variable. Another indicator of the model's effectiveness was the fact that VIF values fell precisely inside the acceptable range (1-10).

**Table 9:** Detailed information of regression model of online language learners’ behavioral engagement.

Model		Unstandardized Coefficients		Standardized Coefficients	t	Sig.	Collinearity Statistics	
		B	Std. Error	Beta			Tolerance	VIF
2	(constant)	1.410	0.116		12.106	0.000		
	Emotional Support	0.328	0.033	0.284	9.794	0.000	0.652	1.533
	Intellectual Support	0.257	0.031	0.241	8.313	0.000	0.652	1.533

Dependent Variable：Behavioral Engagement.

Based on the Beta coefficients from the detailed model data, the standard regression equation for the behavioral engagement of online language learners was Y = 0.284X_1_ (emotional support) + 0.241X_2_ (intellectual support). Therefore, it can be safely concluded that how actively language learners behaved in the online context was closely related to emotional support and intellectual support from the teacher.

Behavioral engagement of online language learners was primarily driven by two variables, namely perceived emotional support and intellectual support, according to data gathered from semi-structured interviews. The excerpt from interviewee A mentioned that both perceived intellectual support and emotional support could positively affect learners’ behavioral engagement, as the professionalism and kindness of the teacher enabled her to actively participate in class. Besides, online communications between the teacher and students would facilitate after-class English learning, suggesting that perceived social support also influenced learners’ behavioral engagement to a certain degree. From the response of interviewee B, it proved that learners’ behavioral engagement could be enhanced by perceived emotional support. Specifically, timely feedback could fulfil the satisfaction of the students so that they would be more likely to behaviorally engage in the class. However, no obvious effect on behavioral engagement was inspected in Interview C, which was mainly attributed to the low engagement of the participant.

To sum up, results from quantitative and qualitative analysis demonstrated that perceived emotional support and intellectual support were positively correlated with language learners’ behavioral engagement in the online context.

(4) Effect of perceived teacher support upon online language learners’ emotional engagement

The impact of perceived teacher support on online language learners’ emotional engagement was investigated using a stepwise method. As it can be found in Table 10, three models in total were produced through the regression analysis and the significance of each model was confirmed by its p value (<0.05). Model 3 (R^2^ = 0.266) ranked first among all the predictive models, containing intellectual support, emotional support and social support, which demonstrated that how language learners emotionally engaged in remote education settings had no bearing on their perceptions of instrumental support from the teacher.

**Table 10:** Summary of model^d^ parametric test.

Model	R	R Square	Adjusted R Square	Std. Error of the Estimate	F	Sig.
1	0.476^a^	0.227	0.226	0.603	418.210	0.000
2	0.512^b^	0.262	0.261	0.589	252.999	0.000
3	0.517^c^	0.267	0.266	0.587	173.253	0.000

a . Predictors: (constant), Intellectual Support.

b . Predictors: (constant), Intellectual Support, Emotional Support.

c . Predictors: (constant), Intellectual Support, Emotional Support, Social Support.

d . Dependent Variable：Emotional Engagement.

In addition, according to Table 11, the p values for each independent variable were lower than 0.05, indicating that these variables could significantly predict the emotional engagement of online language learners. Further proof of the model’s effectiveness came from the fact that VIF values were exactly inside the acceptable range.

**Table 11:** Detailed information of regression model of online language learners’ emotional engagement.

Model		Unstandardized Coefficients		Standardized Coefficients	t	Sig.	Collinearity Statistics	
		B	Std. Error	Beta			Tolerance	VIF
3	(constant)	0.596	0.137		4.346	0.000		
	Intellectual Support	0.371	0.036	0.305	10.178	0.000	0.572	1.747
	Emotional Support	0.242	0.042	0.184	5.750	0.000	0.505	1.981
	Social Support	0.141	0.044	0.104	3.228	0.001	0.493	2.028

Dependent Variable：Emotional Engagement.

The standard regression model equation, Y = 00.305X_1_ (intellectual support) + 0.184X_2_ (emotional support) + 0.104X_3_ (social support), could be derived based on the data in Table 11. As is shown in the equation, perceived intellectual support dominated in having positive effects on the extent to which learners were emotionally engaged in distant language education, closely followed by emotional support and social support.

Data acquired from semi-structured interviews also implied that language learners’ perceived intellectual support, emotional support and social support had a significant impact on their emotional engagement. From interviewee A’s perspective, the teacher’s knowledge reservoir served as a catalyst for learners’ emotional engagement as the information gap intrigued students greatly, and timely praises also inspired the learners. Additionally, conducting various interactive activities was conducive to promoting emotions, especially in the online education settings. Interviewee B believed that both perceived social support and intellectual support enhanced her willingness to engage in the online class. For instance, she thought that they got timely feedback from the teacher for the inquiries. It was unfortunate that was interviewee C’s response failed to provide any concrete proof that perceived teacher support was associated with individual engagement. In his response, he provided more facts, while expressed vague and limited personal feelings, which was largely attributed to his low engagement in online classes.

In conclusion, the findings of both quantitative and qualitative analysis demonstrated that, to varying degrees, the emotional engagement of online language learners was affected by their perceptions of emotional support and social support.

(5) Effect of perceived teacher support upon online language learners’ social engagement

Specific impacts of perceived teacher support on the social engagement of distant language learners were explored and results were presented in Table 12. Data from the model parametric test revealed that two models (p < 0.05) developed from the multiple linear regression analysis were considered significant. Model 2 (R^2^ = 0.408) contained two predictors, namely intellectual support and emotional support, which could explain 40.8 % of variance of online language learners’ social engagement. It is worth mentioning that instrumental support and social support had no significant impact on the dependent variable.

**Table 12:** Summary of model^c^ parametric test.

Model	R	R Square	Adjusted R Square	Std. Error of the Estimate	F	Sig.
1	0.611^a^	0.373	0.373	0.428	850.015	0.000
2	0.640^b^	0.409	0.408	0.416	493.912	0.000

a . Predictors: (constant), Intellectual Support.

b . Predictors: (constant), Intellectual Support, Emotional Support.

c . Dependent Variable：Social Engagement.

Based on the data in Table 13, the p values for each independent variable in Model 2 were all less than 0.05, demonstrating that these factors could strongly predict the social engagement of online language learners. The VIF values were within the allowed range, which was another evidence of the model’s efficacy.

**Table 13:** Detailed information of regression model of online language learners’ social engagement.

Model		Unstandardized Coefficients		Standardized Coefficients	t	Sig.	Collinearity Statistics	
		B	Std. Error	Beta			Tolerance	VIF
2	(constant)	1.039	0.091		11.397	0.000		
	Intellectual Support	0.454	0.024	0.473	18.753	0.000	0.652	1.533
	Emotional Support	0.244	0.026	0.235	9.313	0.000	0.652	1.533

Dependent Variable：Social Engagement.

With detailed Beta coefficients, the standard regression equation of online language learners’ social engagement could be created as Y = 0.473X_1_ (intellectual support) + 0.235X_2_ (emotional support). According to the equation, perceived intellectual support was the most important factor in determining how students were socially engaged in their distant language studies, followed by emotional support.

Qualitative data obtained from semi-structured interviews also provided proof for the effects of perceived teacher support on language learners’ social engagement. Interviewee A believed that perceived emotional, intellectual and social support together promoted her social engagement. To be specific, interviewee A cared about the feedback from the teacher. When she got praise, she made up her mind to work hard, hoping to get more positive feedback. In addition, she expressed her favour for group work, making her more likely to socially engage in English learning. This was probably attributed to her high engagement in class. Therefore, she was more willing to interact with both the teacher and her counterparts. From the response of interviewee B, with perceived emotional support, she considered feedback or appraisal from the teacher important. It is noteworthy that provided online student-student interactions had certain negative effects on individuals’ social engagement. For students with low social engagement, they might adversely impact their group members by presenting perfunctory participation. In the view of Interviewee C, although the teacher provided sufficient opportunities for interactions, he contended that student-student interactions were not as effective as it appeared to be. In other words, interviewee C provided evidence for the statistical results that perceived social support might not significantly affect learners’ social engagement.

Overall, quantitative and qualitative results highlighted that perceived intellectual support took the lead in positively influencing online language learners’ social engagement, while emotional support came in second.

## 5 Discussion

The current study committed to exploring the situation of perceived teacher support and learner engagement, and investigating the effects of perceived teacher support on online language learners’ engagement. The major findings would be discussed regarding three research questions.

(1) Online language learners’ engagement

From the statistical analysis results mentioned above, it can be safely concluded that online language learners’ engagement was generally at an intermediate level. However, there is a long way to reach the advanced level. The conclusion drawn from this research is in line with some previous research findings[10,30,51,[77], [78], [79]]. In other words, most of the learners were found to be moderately engaged in online education settings, which implied that they have already adapted themselves to the new environment. Undoubtedly, online education is far more complicated than traditional education from the root, and achieving high online engagement can be tricky. This is probably one of the reasons that online learners have stagnated at the intermediate engagement level. However, it is worth mentioning that the status quo of language learners’ engagement in e-learning environments in this research is inconsistent with some research findings[55,65] which reported a high engagement level. One of the presumable reason may lie in different sample sizes, disciplines, ages and so on.

Additionally, the current research revealed that emotional engagement was the least engaged dimension, while social engagement ranked the highest in the list, closely followed by behavioral engagement and cognitive engagement, which indicated that learners were still able to socially, cognitively and behaviorally engage in online classes regardless of insufficient emotional engagement. To put it in another way, online learners participated in the class in a relatively passive state. It drops a hint to engagement improvement that sufficient emotional engagement may bring more engagement in other dimensions. Similar patterns can be found in previous research. For instance, Fredricks, Filsecker and Lawson [14] reported that math learners had less emotional engagement in class than behavioral, cognitive and social engagement. Even in three-dimension-model-related (cognitive, behavioral and emotional engagement) research, it was also found that online learners scored the lowest in emotional engagement, while they behaviorally engaged in the class with more effort[10,77,80,81]^.^

With regard to difference analysis, this research suggested that there was no significant difference between males and females in terms of online language learners' engagement, which is in line with previous studies[43,77]. One possible reason for this may result from the uneven gender proportion. Nevertheless, the finding is inconsistent with the conclusions drawn by some studies. For instance, Korlat et al. [50] contended that girls had more online learning engagement than boys. Lietaert et al. [82] also found that boys were less engaged in class than their counterparts. Based on the divergent conclusions, gender difference still is a potential factor influencing individuals’ learning engagement. Besides, this study found no significant difference in online language learners’ engagement among participants from different disciplines, which is accord with the study done by Liu, Zhang and Liu [77]. Notably, some previous studies even didn’t take discipline differences into consideration[43,65].

(2) Perceived teacher support

The statistical analysis revealed that overall online language learners’ perceived teacher support was relatively close to the upper level, which illustrated that most students could perceive adequate support from the teacher during online learning in spite of the spatial distance. This finding is consistent with some previous studies[28,42,50]^.^ However, some researchers disagreed with this conclusion, as they found that online learners perceived teacher support at a medium level[47,77]. One possible reason for this may be attributable to the frequent shift to distance education during the COVID-19 pandemic. Consequently, both educators and learners eventually adapted to online learning so that teachers could be more mindful of providing appropriate assistance and students could be more sensitive to teacher support.

In addition, social support was reported as the least perceived teacher support compared with the other three dimensions, while perceived intellectual support ranked first, closely followed by emotional and instrumental support. It can be inferred that most of the teachers were relatively familiar with distance education and capable of delivering sufficient knowledge support to learners online. Nevertheless, there is still potential for enhancing social interactions for students. This conclusion aligns with the findings in previous studies[28,42]. Language teaching is highly interactive. Under the circumstances of online education, spatial distance may prevent teachers from precisely recognizing learners’ social needs, hence offering insufficient social support.

Based on the difference analysis result, this study found that there was no notable distinction between male and female online language learners in terms of overall perceiving teacher support, which echoed with previous literature [43]. However, there are differing opinions among researchers regarding how the two genders perceived teacher support. For instance, in the research done by Lietaert et al. [82], it was stated that boys perceived less teacher support than girls. In a word, gender difference is still a controversial variable concerning perceived teacher support. Moreover, this study also revealed that there was no significant difference in perceived teacher support among participants from various disciplines. One possible reason for this may be attributable to the fact that each English class is made up of students from different majors at University X.

(3) Effects of perceived teacher support on online language learners’ engagement

According to the Pearson correlation analysis results, the correlation coefficient represented a moderate correlation between overall perceived teacher support and overall online language learners’ engagement. This finding can be well explained with the help of the bio-ecological model of influences on student engagement [58]. It is believed that learners’ engagement would be affected by various external factors which can be further classified as the macro-, exo-, meso- and micro-system ranges and among which teachers play a vital role at the micro-system level. Besides, social support theory claims that teacher support, along with peer support, parents support and so on, is a significant and distinct constituent of social support, which functions as the main source of support that learners can gain at school. Thus, overall perceived teacher support can positively predict overall learners’ engagement.

Furthermore, multiple linear regression analysis results indicated that intellectual support was the most significant factor in predicting overall online language learners’ engagement, closely preceded by emotional support, social support and instrumental support. To put another way, online language learners might actively participate in the class to a greater extent if they perceived a higher level of intellectual support from their teacher. The finding is remarkably in line with the research conducted by Liu, Zhang & Liu [77]. The study revealed that teacher’s autonomy support took the lead in affecting overall online learners engagement compared with cognitive support and emotional support. Autonomy support shares some similarities with intellectual support from the root since both of them concerns teaching strategies. One possible reason for intellectual support dominating in positively predicting online learner engagement is that online settings have a higher demand for content knowledge and pedagogical knowledge to draw the attention of learners because teachers can hardly capture students’ states as they do in face-to-face class. In addition, emotional support came in second in both studies, which further verified its significant effects on learner engagement.

In terms of cognitive engagement, the results of multiple linear regression analysis argued that intellectual support exerted the greatest influence compared with other kinds of supports. In other words, if online language learners could perceive more intellectual support from the teacher, they are more likely to cognitively engage in the class. This conclusion shares consistency with previous studies. The methods and strategies adopted by the teacher well guided learners in online learning, which further facilitated their cognitive engagement as they could employ cognitive strategies as well as meta-cognitive strategies to process learning materials and dispel the thoughts that may place hindrance on concentration and be more engaged in class cognitively[83,84]. Furthermore, the results from semi-structured interviews resonated with the data from Muir et al. [61], which provided qualitative evidence to the fact that perceived teaching methods and strategies were beneficial to online language learners’ engagement. With regard to specific strategies influencing learner engagement, Housand & Reis [85], for instance, proved that modelling was the facilitator for engaging learners in tasks, enlightening them in response to high-order thinking questions and developing conceptual abilities as well as application, thus enhancing cognitive engagement. Possible reasons for the results may be analyzed from the definitions of the two terms. Cognitive engagement is closely related to the use of learning strategies as can be identified from the concept. Teachers hold a pivotal role in class not only for imparting knowledge, but also for introducing methods and approaches that could benefit learner’ lifelong study. Thus, perceived intellectual support and cognitive engagement share many similarities from the root, which ensures the correlation between the two variables.

As for behavioral engagement, perceived emotional support and intellectual support could exert positive impact, while instrumental support and social support failed to make contributions. The dominant influence of emotional support was also confirmed, which is consistent with established studies. Havik & Westergård [48] verified that emotional support from the teacher could positively and significantly predict learners’ behavioral engagement. The more emotional support learners perceive, the harder they tend to behave. It is argued that feedback from the educator does make a difference for learners’ behavior engagement[86,87]. When positive learning behavior is reinforced by praise, learners are willing to behaviorally engage in class, which highlights the function of emotional support. In this regard, Tas [8] mentioned that equal treatment and attention for students could grow their participation in learning. One possible reason for these findings can be inferred that behaviors are governed by intrinsic motivation of individuals. As for intellectual support, Goss, Sonnemann and Griffiths [87] also mentioned the challenges that teachers encountered in terms of fostering learning behavior, entailing the knowledge concerning effective approaches and strategies and how to utilize them appropriately, which underscored the significance of intellectual support provided by the instructor.

With regard to emotional engagement, statistical analysis indicated that perceived intellectual support had the strongest influence, while perceived emotional support and social support followed afterwards. Obviously, perceived instrumental support had no association with the extent to which learners emotionally engaged in online language class. Presumably the reason is that the instructor may find it hard to recognize the supporting needs and provide timely help in online settings, as learners may be reluctant to state their difficulties. The findings are in accordance with previous studies[48,56]. Newmann, Wehlage & Lamborn [88] claimed that the approach to present the topics and activities, the former experience of the teacher and other relevant pedagogical methods were more likely to arouse the interest of the learner than the teaching content itself. This identifies that intellectual support from the teacher can facilitate learners’ emotional engagement. With regard to the predicting role of perceived emotional support, Skinner & Belmont [89] stated that if the teacher expressed positive affection towards students, they would feel more enthusiastic and more optimistic. A possible interpretation for this may be that emotionally supportive instructors would enhance individuals’ self-efficacy which is entwined with learner engagement based on the aforementioned literature.

For social engagement, the findings implied that intellectual support again took the lead and emotional support came in second, while instrumental support and social support failed to predict the extent to which learners were socially engaged in online class. The findings echo with previous research to some extent. Klem & Connell [90] revealed that if the instructor could provide a positive and well-organized learning environment, the students would be more willing to engage in class. Therefore, methods and strategies adopted by teachers, teaching experience and resources accumulated by teachers, and respect, feedback and encouragement provided to learners are of great significance for building a harmonious class environment.

## 6 Conclusion

The current study examined the status quo of online language learners’ engagement (cognitive, behavioral, emotional and social engagement) and perceived teacher support (emotional, intellectual, instrumental and social support), and the associations between the two variables. The findings are as follows: (1) Online language learners’ engagement was generally at an intermediate level, and emotional engagement was the least engaged dimension. In addition, there was no significant difference in online language learners’ engagement in terms of demographic factors. (2) Overall online language learners’ perceived teacher support was relatively close to the upper level. Perceived social support was reported as the least perceived teacher support compared with the other three dimensions, while perceived intellectual support ranked first. Besides, perceived teacher support did not differ significantly between genders or disciplines. (3) Perceived teacher support could positively predict online language learners’ engagement. Specifically, perceived intellectual support dominated in positively impacting overall engagement, cognitive engagement, emotional engagement and social engagement, whereas perceived emotional support had the strongest influence on online language learners’ behavioral engagement.

## 7 Implications

The findings of the current study have yielded both theoretical and practical implications for literature on learner engagement and language teaching practice respectively. With regard to theoretical implications, first of all, this study adds empirical evidence to existing studies on learner engagement, responding to the research gap of both online language learners’ engagement and their perceived teacher support as well as verifying the predictive role of perceived teacher support in online language teaching scenario. Secondly, the survey-based design contributes reliable and valid ways to measure how engaged online learner are and how much support they perceive from the teacher. The domain-specific indicators make it easier to measure the variables as domain-general scales popularize among previous relevant literature. Thirdly, previous literature mostly chose quantitative methods, specifically survey[42,65,91], while some researchers took qualitative approaches[10,14]. Sequential mixed-methods adopted in this research can help better address research questions and provide deeper understanding for the results, as quantitative and qualitative methods combined show a more comprehensive picture of the research results, which goes beyond the sum of the two separate parts. In terms of practical implications, first of all, according to the statistical results, instructors should pay more attention to how to provide more intellectual support to online learners as it positively correlated with several dimensions of learner engagement. Specifically, the teacher should entail solid professional competence, be it academic or pedagogical, and be ready for any knowledge updates to satisfy learners’ needs. Furthermore, teachers are supposed to display emotional outflow and can show more rapport, care, encouragement and recognition for students especially under the condition of time and space barriers, since emotionally supportive educators enable to motivate online learners to engage in class activities.

## 8 Limitations and suggestions for future research

There are some limitations for this research. First of all, the tool for measuring online language learner engagement and perceived teacher support is the scale. Although survey is the most commonly used method, subjective data is accompanied by uncertainty since it is unknown that whether learners have clear perceptions about their learning and teacher support, which may affect the accuracy of the results. In addition, learner engagement is inherently dynamic and scales do not allow for real-time understanding about the engagement level. Future research can look for more comprehensive and advanced ways to accurately and timely identify how learners are engaged during learning process and to what extent they perceive teacher support. For instance, Bonner et al. [92] already developed an online application to measure real-time English learner engagement in the online context. Secondly, participants in this study exhibited good English proficiency and were first-year undergraduates. However, it is necessary to acknowledge that these characteristics may restrict the extent to which the findings may be applied to a broader context. Moreover, the current study does not take the fact into consideration that participants taught by different teachers may have potential impacts on the measurement of learner engagement and perceived teacher support due to different teaching styles and strategies, as it is hard to control this variable with large sample size. Apart from that, participants recruited in this study failed to take non-stop online courses, as University X temporarily restored offline courses for several days. Therefore, this may shed certain effects on the research results to some extent. It is worth mentioning that data collected from semi-structure interviews found that online language learners also perceived other support from the teacher (As interview B mentioned, ‘despite the teacher was tested positive for COVID-19, had a high fever and coughed a lot, she still gave lectures to us, and this was very touching’), which further facilitates their engagement. Therefore, future studies may be enlightened by this unexpected finding and explore the constituents of perceived teacher support crosswise due to its malleability. Additionally, mediators for the impact studies can be explored in future research.

## Ethics statement

This study was reviewed and approved by College of Foreign Languages, Nanjing University of Aeronautics and Astronautics ethics committee. All participants provided Written Informed Consent to participate in the study.

## Data Availability statement

The datasets generated and/or analyzed during the current study are available from the corresponding author on reasonable request.

## CRediT authorship contribution statement

**Lingqi Fan:** Writing – review & editing, Writing – original draft, Validation, Resources, Project administration, Methodology, Investigation, Formal analysis, Data curation, Conceptualization.

## Declaration of competing interest

The authors declare that they have no known competing financial interests or personal relationships that could have appeared to influence the work reported in this paper.
