# Supplementary material for: Effects of perceived teacher support on online language learners’ engagement
Source: Heliyon. 2024 Aug 7;10(15):e35679. doi: 10.1016/j.heliyon.2024.e35679 (PMC11336843; doi:10.1016/j.heliyon.2024.e35679)
Supplement: Multimedia component 2 [file mmc2.pdf]

Questionnaire on Online Language Learners' Engagement and Perceived Teacher Support

Instructions: Dear students, you have been invited to take part in a study about online language learners' engagement and perceived teacher support. Please read the following information as carefully as possible before you decide whether to take part in this study. It will help you understand the study and the procedures of the study, and the possible risks and discomforts of participating in the study. Please read it carefully and if you have any questions, please do not hesitate to ask the researcher.

1. Research Project title

Effects of Perceived Teacher Support on Online Language Learners' Engagement

2. Research process

The questionnaire requires you to fill in the information according to your own actual situation. Please response to each item in line with your own opinions and blacken the item circle you choose. In order to ensure the quality of the questionnaire, please read the items carefully and do not omit any items. Also, please do not share the questionnaire with others. Besides, please note that there is a chance for you to engage in further semi-interview session in the later stage of the research.

3. Risks, discomforts that may arise from participating in this study

We will not disclose any of your information and there is no risk of disclosing it to others, please feel free to fill in the questionnaire.

4. Confidentiality of personal information and records

If you decide to participate in this study, your personal information will be kept strictly confidential all the way through this study. The researcher is required to keep your identifying information confidential and can ensure that your identifying information will not be disclosed to members outside the researcher unless you give your permission.

5. You may voluntarily choose to participate in or withdraw from the study

Whether or not you participate in the study is entirely up to you. If you have any questions about the study, you may ask the researchers. You may also refuse to participate in the study or withdraw from the study at any time during the study without affecting the relationship between you and the researchers or your rights.

As a research subject, you have a duty to state the facts. If you do not comply with this duty, the researchers may terminate your continued participation in this study.

Informed Consent

I have read the above description of this study. I am aware of the possible risks of participating in this study, my participation in the study is voluntary and I confirm that I have had sufficient time to consider this and understand that:

1. I can ask the researcher for more information at any time.
2. I can withdraw from this study at any time without discrimination or retaliation.
3. If I do not follow the study plan and complete it carefully, the researchers will terminate my continued participation in this study.

Finally, I have decided to agree to participate in this study.

○Yes ○No

My gender is _____.

○Male

○Female

I am from the discipline of _____.

○Agriculture

○Art

○Economics

○Education

○Engineering

○Law

○Literature

○Management

○Medicine

○Science

My student ID is________.

Online Language Learners' Engagement Scale

Cognitive Engagement

1. I try to connect learning materials with previous knowledge in online English class.

   ○completely disagree ○disagree ○unsure ○agree ○completely agree

2. I try to set examples to better understand the important concepts in online English class.

   ○completely disagree ○disagree ○unsure ○agree ○completely agree

3. I put forth effort to figure out different ways to solve the problems encountered in online English class.

   ○completely disagree ○disagree ○unsure ○agree ○completely agree

4. I try to figure out how the knowledge learned in online English class might be used in the real world.

   ○completely disagree ○disagree ○unsure ○agree ○completely agree

5. I reflect on my understanding of the feedback that I get in the online English class.

   ○completely disagree ○disagree ○unsure ○agree ○completely agree

Behavioral Engagement

1. I try my best even when it is hard in online English class.

   ○completely disagree ○disagree ○unsure ○agree ○completely agree.

2. I complete the class-related practice/homework.

   ○completely disagree ○disagree ○unsure ○agree ○completely agree

3. I learn English outside of the online English class.

   ○completely disagree ○disagree ○unsure ○agree ○completely agree

Emotional Engagement

1. I look forward to attending online English class.

   ○completely disagree ○disagree ○unsure ○agree ○completely agree

2. I enjoy learning new things in online English class.

○completely disagree ○disagree ○unsure ○agree ○completely agree

3. I often like doing challenging tasks in online English class.

   ○completely disagree ○disagree ○unsure ○agree ○completely agree

4. I enjoy sharing my opinions/experiences with the teacher and classmates in online English class.

   ○completely disagree ○disagree ○unsure ○agree ○completely agree

5. I enjoy discussing course concepts/issues/ideas with my peers during online discussions.

   ○completely disagree ○disagree ○unsure ○agree ○completely agree

Social Engagement

1. I try to help the students who meet with difficulties when learning online.

   ○completely disagree ○disagree ○unsure ○agree ○completely agree

2. I like working with others to finish tasks in online English class.

   ○completely disagree ○disagree ○unsure ○agree ○completely agree

3. I try to do the tasks with classmates who can help me.

   ○completely disagree ○disagree ○unsure ○agree ○completely agree

4. I am very concerned about the comments and suggestions from my teacher or classmates.

   ○completely disagree ○disagree ○unsure ○agree ○completely agree

Online Language Learners' Perceived Teacher Support Scale

Emotional Support

1. Teacher respects my response and feedback in online English class.

   ○completely disagree ○disagree ○unsure ○agree ○completely agree

2. Teacher encourages me to state my own opinions in online English class.

   ○completely disagree ○disagree ○unsure ○agree ○completely agree

3. The teacher offers me suggestions on learning English and helps me establish my confidence in online English class.

○completely disagree ○disagree ○unsure ○agree ○completely agree

4. Teacher praises and recognizes me for making progress or completing tasks in online English class.

   ○completely disagree ○disagree ○unsure ○agree ○completely agree

5. Teacher is friendly to me in online English class.

   ○completely disagree ○disagree ○unsure ○agree ○completely agree

Instrumental Support

1. When I have problems with English in online class, I get good help and guidance from my English teacher.

   ○completely disagree ○disagree ○unsure ○agree ○completely agree

2. Teacher provides me with useful guidance and help in online English class.

   ○completely disagree ○disagree ○unsure ○agree ○completely agree

3. Online English class platform is user-friendly.

   ○completely disagree ○disagree ○unsure ○agree ○completely agree

Intellectual Support

1. Teacher gives lesson with clear pronunciation, fluent expression in online English class.

   ○completely disagree ○disagree ○unsure ○agree ○completely agree

2. Teacher provides me with sufficient learning resources in online English class.

   ○completely disagree ○disagree ○unsure ○agree ○completely agree

3. Teacher organizes course content in a reasonable way.

   ○completely disagree ○disagree ○unsure ○agree ○completely agree

4. Teacher is equipped with broad knowledge that broadens my horizons and motivates me to learn.

   ○completely disagree ○disagree ○unsure ○agree ○completely agree

5. Teacher asks inspiring questions in online English class.

   ○completely disagree ○disagree ○unsure ○agree ○completely agree

6. Teacher is familiar with online teaching.

○completely disagree ○disagree ○unsure ○agree ○completely agree

Social Support

1. Teacher uses social media to communicate with us after class, and I establish harmonious rapports with the teacher.

   ○completely disagree ○disagree ○unsure ○agree ○completely agree

2. Teacher well guides me to group learning.

   ○completely disagree ○disagree ○unsure ○agree ○completely agree

3. Teacher notices my statements in the chat box or on other platforms, and gives response.

   ○completely disagree ○disagree ○unsure ○agree ○completely agree

4. Teachers would discuss with us about course-related stuff in online class.

   ○completely disagree ○disagree ○unsure ○agree ○completely agree

5. I would love to participate in the activities organized by the teacher.

   ○completely disagree ○disagree ○unsure ○agree ○completely agree
